# Case report: Carcinosarcoma of uterus in nulliparous women

**DOI:** 10.3389/fonc.2024.1472416

**Published:** 2024-12-11

**Authors:** Feiyue Sun, Xuelei Li, Luyao Kang, Yiran Wang, Hongyu Li, Hai Zhu

**Affiliations:** Gynecologic Oncology, The Third Affiliated Hospital of Zhengzhou University, Zhengzhou, China

**Keywords:** carcinosarcoma of uterus, nulliparous women, malignant mixed Müllerian tumor, endometrial carcinoma, case report

## Abstract

**Background:**

Uterine carcinosarcoma (UCS), or malignant mixed Müllerian tumor, is a cancer that include both carcinomatous and sarcomatous components, resembling endometrial carcinoma. A 55-year-old woman was admitted to the hospital with postmenopausal vaginal bleeding. Gross examination of the specimen revealed brittle tissue in the fundus and the left wall of the endometrium. Postoperative pathology revealed a mixture of well-differentiated endometrioid adenocarcinoma and osteosarcoma. The patient was never given birth, which may be relevant to the diagnosis. Literature review suggests that being nulliparous may be a significant risk factor for developing uterine carcinosarcoma.

**Case description:**

In December 2023, a 55-year-old female patient was admitted to the hospital with postmenopausal vaginal bleeding. Hysteroscopic surgery was performed, and the postoperative pathology showed endometrial cancer accompanied by ossified tissue with necrosis. The immunohistochemical results indicated positive Estrogen receptors (ER), positive Progesterone receptors (PR), ki67 positivity at 70%, negative PTEN, mutated positive p53, focal positive Pax-8, positive SATB2, positive Cytokeratin 7 (CK7), positive EMA and positive Vimentin (Vim). The patient was diagnosed with Uterine carcinosarcoma. On December 18, 2023, the patient underwent partial vaginal resection, bilateral salpingo-oophorectomy, pelvic lymph node dissection, and a sub-extensive laparoscopic hysterectomy. Postoperatively, the patients received radiotherapy and four cycles of chemotherapy in the DC regimen. As of July 2024, laboratory and impact test results showed no tumor recurrence. The patient’s disease-free survival (DFS) was seven months.

**Conclusion:**

The rate of childless in patients with uterine carcinosarcoma is at a high level.

## Introduction

Uterine carcinosarcoma (UCS), also known as malignant mixed Müllerian tumor, is a rare type of cancer that consists of both carcinomatous and sarcomatous components, and it presents similarly to resembling endometrial carcinoma (1). UCS is rare, representing 2% to 5% of malignant tumors in the uterine corpus (2). However, it accounts for 16% of deaths caused by uterine malignancies and is associated with a poor prognosis (3). In comparison to women with endometrioid or high-grade serous uterine malignancies, those diagnosed with UCS have significantly lower survival rates (4).

Diagnosing uterine carcinosarcoma requires pathology, but there is currently a lack of early and reliable diagnostic techniques (5). A key feature of UCS is biphasic histology, which includes both sarcomatous (mesenchymal) and carcinomatous (epithelial) components (6). The epithelial component is the main factor affecting how tumors behave biologically. Surgical resection, followed by postoperative therapy, remains the best curative option for UCS (7). Studies have shown that chemoradiation improves clinical outcomes more effectively than chemotherapy or radiotherapy alone (8). Studies indicate that ethnicity influences both progression-free survival and overall survival in uterine carcinosarcoma (9). The pathogenesis of uterine carcinosarcoma remains unclear; however, its development has been associated with factors such as obesity, prolonged use of tamoxifen or estrogen, pelvic radiation therapy, diabetes mellitus, and an early age at menarche (10). We will describe a case of UCS that was confirmed to be high-grade endometrioid carcinoma with an osteosarcoma component. Our retrospective analysis revealed that the patients were unmarried and childless. This case report represents the first documented instance of uterine carcinosarcoma in unmarried and nulliparous patients. Based on our findings, we believe that the risk of developing uterine carcinosarcoma is relatively high in women who have never given birth (nulliparous).

## Case description

**Figure 1:**
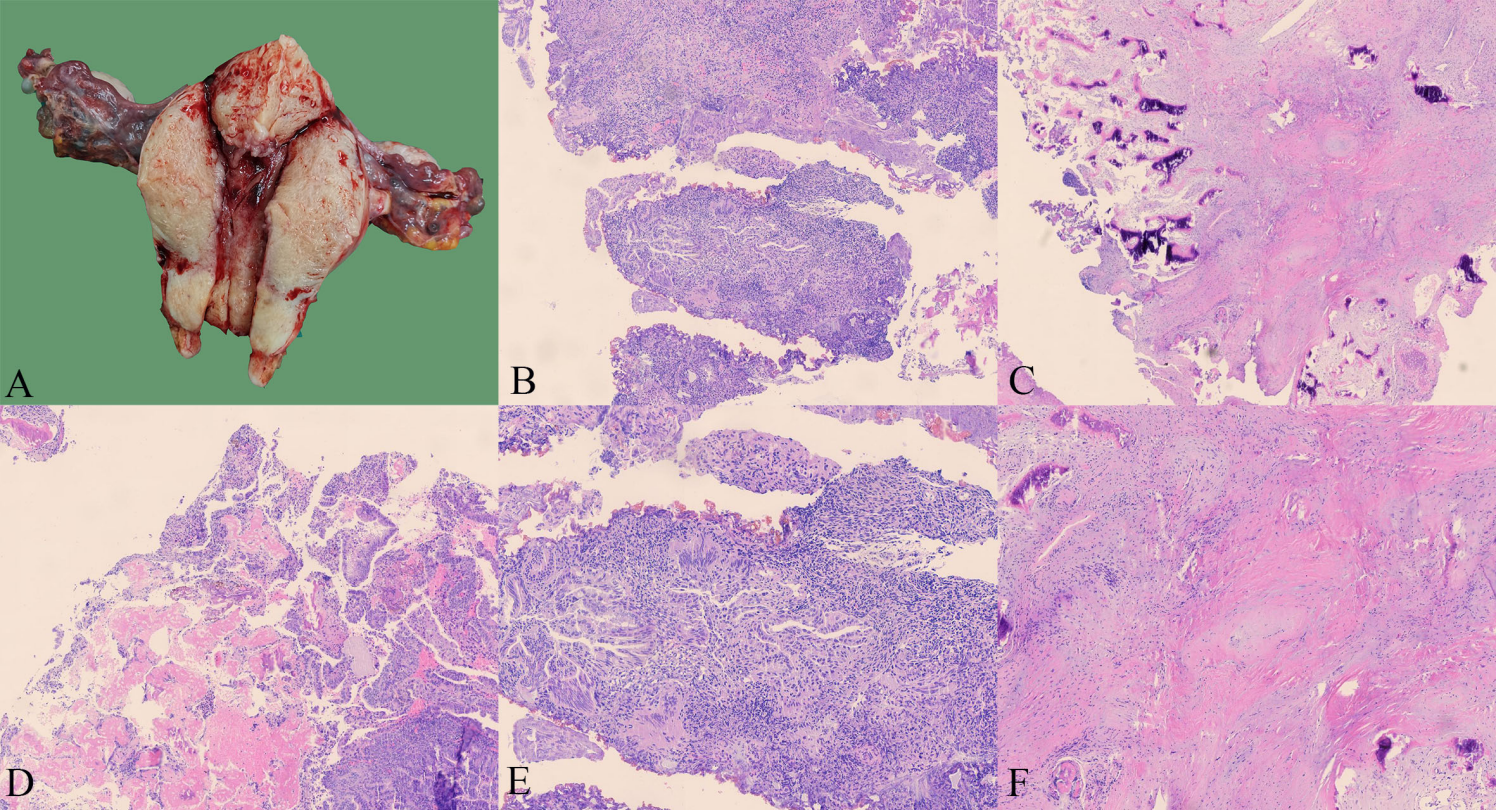
The patient’s intraoperative condition and pathological results **(A)** Ectomy of the uterus shows that the fundus and the left wall endometrium can see brittle tissue **(B)** Well-differentiated endometrioid adenocarcinoma (HE; x40) **(C)** Osteosarcoma area (HE; x40) **(D)** Mixed area of well-differentiated endometrioid adenocarcinoma and osteosarcoma (HE; x40) **(E)** Well-differentiated endometrioid adenocarcinoma (HE; x100) **(F)** Osteosarcoma area (HE; x100).

**Figure 2:**
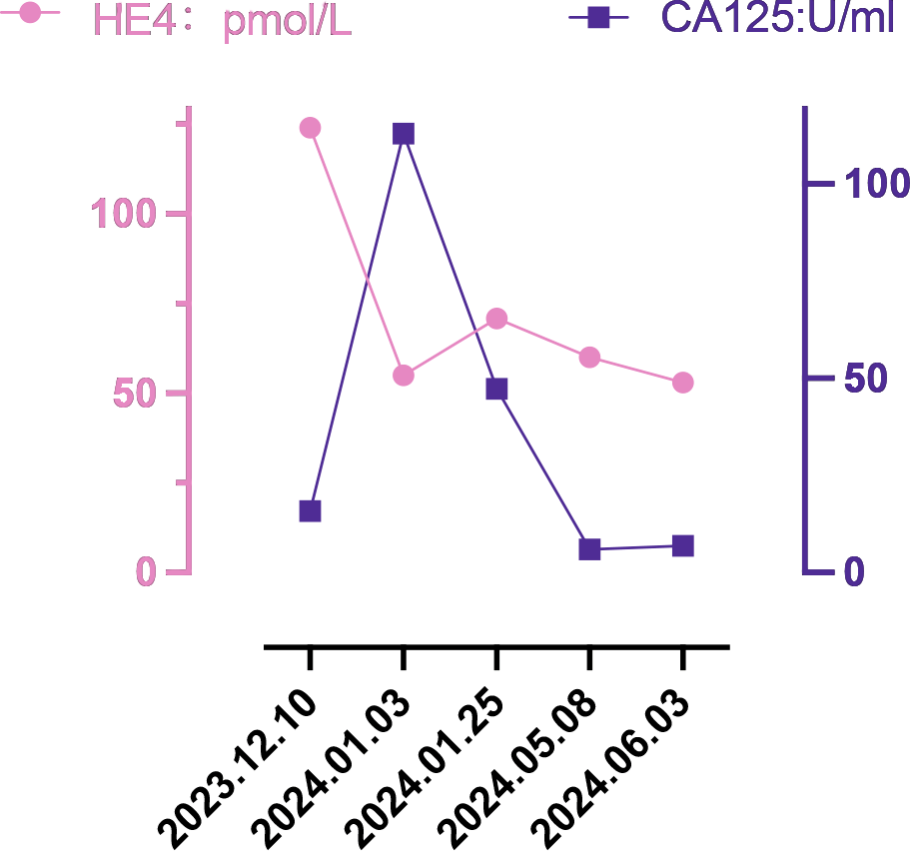
Changes in tumor markers.

**Figure 3:**
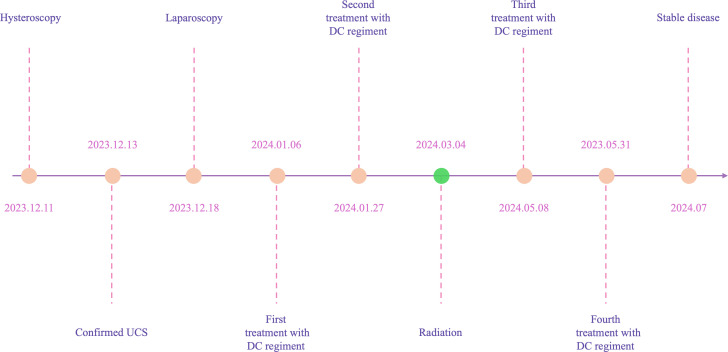
A timeline with relevant data from the episode of care.

In December 2023, a 55-year-old woman, postmenopausal for three years, experienced moderate vaginal bleeding that resembled menstrual flow. The bleeding was bright red and did not contain blood clots. To date, she has experienced a small amount of vaginal bleeding, accompanied by occasional vague pain in the lower abdomen. An ultrasound examination one day ago revealed the uterine cavity measuring approximately 25 mm by 15 mm, with a poorly defined boundary. On December 11, 2023, a hysteroscopy and resection of uterine cavity mass were performed. The pathological diagnosis confirmed UCS, and the epithelial component immunohistochemical results are detailed below: Estrogen receptors (ER) (+), Progesterone receptors (PR) (+), Proliferating Cell Nuclear Antigen K-67 (Ki67) (+,70%), Phosphatase and Tensin Homolog (PTEN) (-), p53 (+, wild type pattern), Paired Box 8 (Pax-8) (+, focal), Special AT-rich Sequence-binding Protein 2 (SATB2) (+), Cytokeratin Pan (CK-Pan) (+), Cytokeratin 7 (CK7) (+), Epithelial Membrane Antigen (EMA) (+), Vimentin (Vim) (+).The patient was diagnosed with uterine carcinosarcoma. On December 18, 2023, the patient underwent several surgical procedures, including a partial vaginal resection, bilateral salpingo-oophorectomy, pelvic lymph node dissection, and a sub-extensive laparoscopic hysterectomy (**Figure 1A**). Postoperative routine pathology confirmed the diagnosis of uterine carcinosarcoma (**Figure 1D**). The pathology report identified two components: the epithelial component is high-grade endometrioid carcinoma (**Figures 1B, E**), while the sarcoma component is a heterologous osteosarcoma (**Figures 1C, F**). The tumor is classified as stage IC. The patient underwent two cycles of the DC regimen, which included Docetaxel (75 mg/m2 on day 1 every three weeks) and Carboplatin (AUC6), from January 6 to January 27. Radiation therapy was then administered. After completing radiotherapy treatment, the patient received two additional cycles of chemotherapy from May 13 to June 5, 2024, and their condition remained stable. Prior to surgery, the HE4 index was quite high; during surgery, it dramatically dropped; and with chemotherapy and radiation therapy, it stayed at a normal level. Interestingly, the CA125 index was within the normal range prior to surgery, rose during the procedure, fell following chemotherapy and radiation, and then returned to normal levels (**Figure 2**). The patient’s complete treatment timeline is to enhance understanding of the diagnostic and therapeutic process (**Figure 3**).

Upon reviewing the patient’s medical history, we found that she was never given birth. In April 2024, we conducted a literature search using the terms “carcinosarcoma,” “malignant mixed mesodermal tumor,” and “malignant mixed Mullerian tumor.” Our goal was to determine whether childless is a high-risk factor for developing uterine carcinosarcoma. We defined childless as never having given birth, and aligned with this definition in retrospective studies, and results that do not meet this definition will be discarded. The search yielded seven relevant studies (**Table 1**) (9–15). We found that seven literatures contained 1493 patients with uterine carcinosarcoma from six countries: Thailand, India, Japan, the United States, Italy, and Portugal.

**Table 1:** Clinical data of patients with uterine carcinosarcoma from the literature.

Author	Year of publication	Age (Mean ± SD)	Region	Number of cases of uterine carcinosarcoma	Number of nulliparous cases
Abhilasha Goyal (9)	2023	59.57 ± 11.78	India	20	6
Koji Matsuo (10)	2019	≥60:583^a^ <60:294	Japan/United States	877	139
Silvana Grasso (11)	2016	65.99 ± 10.25	Italy	39	6
Rita Luz (12)	2016	71.29 ± 15.35	Portugal	42	7
Kenichi Harano (13)	2016	64.94 ± 9.46	Japan	475	60
Chalermrat Potikul (14)	2016	60.3 ± 9.20	Thailand	25	13
Ahitagni Biswas (15)	2012	59.66 ± 10.92	India	15	0

a Koji Matsuo (10)’s study was a multicenter study of 42 medical centers in Japan and the United States, and the original text only provided a stratified distribution of patient age, so the mean and standard deviation of patient age could not be obtained.

## Discussion

Uterine carcinosarcoma primarily consists of epithelial and mesenchymal components (16). Approximately two-thirds of the epithelial components are serous carcinomas, while one-third are endometrioid carcinomas; Most mesenchymal components are high grade sarcomas. Homologous UCS consists of sarcoma components derived from uterine tissue (endogenous differentiation), including endometrial stromal sarcoma, undifferentiated sarcoma, fibrosarcoma, or leiomyosarcoma, or a combination of these. In contrast, heterologous UCS includes sarcoma components from outside the uterine tissue (exogenous differentiation), such as rhabdomyosarcoma and chondrosarcoma; however, osteosarcoma and liposarcoma are rare (3). In this case, uterine carcinosarcoma includes components of both endometroid carcinoma and osteosarcoma, which is relatively rare. UCS is a rare and aggressive form of high-grade endometrial cancer, which has a poor prognosis even when treated with various methods, including surgery, platinum-based chemotherapy, radiotherapy, and radiotherapy. Survival statistics for UCS are concerning: the median overall survival time is under two years, and the five -year overall survival rate was less than 30%. Even in patients with early-stage disease, the five-year recurrence rate stands at 45%, while the associated mortality rate over the same period is 50% (16–19). Recently, classifying endometrial cancer by the presence of specific markers—such as polymerase-epsilon (POLE) exonuclease domain mutations (EDMs), protein 53 (p53) immunohistochemistry, and mismatch repair (MMR) proteins has become a crucial tool in its treatment (20). This approach enhances the management and precision of endometrial cancer treatment (21). The latest imaging test shows no clear signs that the cancer has spread or recurred. **Figure 2** illustrates that the patient’s preoperative HE4 index was significantly elevated at 124 pmol/L. After surgery, this value dropped to 55 pmol/L, and following four cycles of chemotherapy and radiotherapy, ultimately returning to normal. Initially normal, the preoperative CA125 index briefly increased after surgery, then gradually decreased post-chemoradiotherapy, and finally stabilized at a normal level. The development of this cancer is linked to several aetiological variables, including radiation exposure to the pelvis, obesity, nulliparity, and exposure to exogenous estrogen or the human papilloma virus (22). Current mainstream research focuses on three main areas: obesity, radiation exposure to the pelvic, and racialized risk factors (23). In the six countries we studied, the mean age of onset of uterine carcinosarcoma for patients in Asian countries (India, Thailand, and Japan) ranged from 59.57 to 64.94 years (the mean value was calculated by converting the sample size, minimum, median, and maximum values) (24). This is generally lower than the mean ages in European countries (Italy, Portugal), which were 65.99 and 71.29 years, respectively. Out of the 1,493 cases we investigated, 231 were childless, accounting for 15.47%. The global female childless rate varied from 3.5% to 16.7% (25), and the childless rate of women with intrauterine carcinosarcoma in our study was at the upper end of this range. In recent years, the desire to have children is declining globally (26, 27), leading to a decline in the number of births. Could this trend affect the incidence of carcinosarcoma of the uterus?

## Data Availability

The original contributions presented in the study are included in the article/supplementary material. Further inquiries can be directed to the corresponding authors.
